# Breast Feeding Increases Vasoconstriction Induced by Electrical Field Stimulation in Rat Mesenteric Artery. Role of Neuronal Nitric Oxide and ATP

**DOI:** 10.1371/journal.pone.0053802

**Published:** 2013-01-14

**Authors:** Javier Blanco-Rivero, Esther Sastre, Laura Caracuel, Miriam Granado, Gloria Balfagón

**Affiliations:** 1 Departamento de Fisiología, Facultad de Medicina, Universidad Autónoma de Madrid, Madrid, España; 2 Instituto de Investigación Sanitaria IdIPaz, Madrid, España; Universidade Federal do Rio de Janeiro, Brazil

## Abstract

**Objectives:**

The aim of this study was to investigate in rat mesenteric artery whether breast feeding (BF) affects the vasomotor response induced by electrical field stimulation (EFS), participation by different innervations in the EFS-induced response and the mechanism/s underlying these possible modifications.

**Methods:**

Experiments were performed in female Sprague-Dawley rats (3 months old), divided into three groups: Control (in oestrous phase), mothers after 21 days of BF, and mothers that had recovered their oestral cycle (After BF, in oestrous phase). Vasomotor response to EFS, noradrenaline (NA) and nitric oxide (NO) donor DEA-NO were studied. Neuronal NO synthase (nNOS) and phosphorylated nNOS (P-nNOS) protein expression were analysed and NO, superoxide anion (O_2_^.–^), NA and ATP releases were also determined.

**Results:**

EFS-induced contraction was higher in the BF group, and was recovered after BF. 1 µmol/L phentolamine decreased the response to EFS similarly in control and BF rats. NA vasoconstriction and release were similar in both experimental groups. ATP release was higher in segments from BF rats. 0.1 mmol/L L-NAME increased the response to EFS in both control and BF rats, but more so in control animals. BF decreased NO release and did not modify O_2_^.–^ production. Vasodilator response to DEA-NO was similar in both groups, while nNOS and P-nNOS expressions were decreased in segments from BF animals.

**Conclusion:**

Breast feeding increases EFS-induced contraction in mesenteric arteries, mainly through the decrease of neuronal NO release mediated by decreased nNOS and P-nNOS expression. Sympathetic function is increased through the increased ATP release in BF rats.

## Introduction

Breast feeding is characterised by strict hormonal control mediated by oestrogen, progesterone, prolactin and oxytocin, associated with increases in gastrointestinal blood flow and cardiac output [1], [2] to provide the udder with nutrients and hormones that regulate milk synthesis and secretion. Some hormones implicated in the lactation process (prolactin, oxytocin) have been reported to alter vascular function [3], [4].

Vascular tone is determined by an equilibrium among several mechanisms, including hormonal, metabolic and neuronal factors. This neural regulation involves sympathetic, cholinergic, nitrergic, peptidergic and/or sensory innervations that are specific to the vascular bed considered [5], [6]. In rat mesenteric arteries, vascular tone is mediated by the integrated action of different neurotransmitters, mainly noradrenaline (NA) and ATP from sympathetic nerve terminals, neuronal nitric oxide (NO) from nitrergic innervation and calcitonin gene-related peptide (CGRP) from sensory nerves [7], . Several experimental and pathophysiological circumstances including ageing, hypertension and diabetes, have been shown to alter the functional role of these components [11], [12], [13].

We have previously reported that sex steroids modulate the synthesis and/or sensitivity to these neurotransmitters in rat mesenteric arteries [14], [15]. Reports of the effects of breast feeding on sympathetic activity are contradictory, with increased and decreased sympathetic tone being described [16], [17], [18]. On the other hand, increased serum nitrite and nitrate concentrations [19] and ROS generation [20] have been reported in breast-feeding mothers. These findings indicate that the functional role of adrenergic and nitrergic innervations could be affected in breast feeding and this would affect peripheral vascular resistance.

Therefore, the aim of this study was to investigate whether breast feeding affects neurotransmitter release or EFS-induced vasomotor response in rat mesenteric arteries, the participation of different innervations in the EFS-induced response and the mechanism/s underlying these possible modifications.

## Methods

### Animals

Female Sprague-Dawley rats (3 months-old) were obtained from the Animal Quarters of the Universidad Autónoma de Madrid and housed in the Animal Facility of the Universidad Autónoma de Madrid (Registration number EX-021U) in accordance with guidelines 609/86 of the E.E.C., R.D. 233/88 of the *Ministerio de Agricultura, Pesca y Alimentación* of Spain, and Guide for the Care and Use of Laboratory Animals published by the United States National Institute of Health [NIH publication No. 85.23, revised 1985]. All experimental procedures involving animal use were approved by the Ethics Committee of the *Universidad Autónoma de Madrid*. Rats were housed during treatment at a constant room temperature, humidity, and light cycle (12∶12 h light-dark) with free access to tap water and fed with standard rat chow *ad libitum*. Rats were divided into three groups: Control (virgin females in oestrous phase), mothers after 21 days of breast feeding (BF), and mothers that had recovered their oestral cycle (After BF, in oestrous phase).

Animals were sacrificed by CO_2_ inhalation; the first branch of the mesenteric artery was carefully dissected, cleaned of connective tissue and placed in Krebs-Henseleit solution (KHS, in mmol/L: NaCl 115, CaCl_2_ 2.5, KCl 4.6, KH_2_PO_4_ 1.2, MgSO_4_. 7H_2_O 1.2, NaHCO_3_ 25, glucose 11.1, Na_2_EDTA 0.03) at 4°C. For protein expression analysis, some arteries were rapidly frozen in liquid nitrogen and kept at −80°C until the day of analysis.

### Vascular Reactivity

The method used for isometric tension recording has been described in full elsewhere [9], [21]. Two parallel stainless steel pins were introduced through the lumen of the vascular segment: one was fixed to the bath wall and the other connected to a force transducer (Grass FTO3C; Quincy, Mass., USA); this, in turn, was connected to a model 7D Grass polygraph. For EFS experiments, segments were mounted between two platinum electrodes 0.5 cm apart and connected to a stimulator (Grass, model S44) modified to supply adequate current strength. Segments were suspended in an organ bath containing 5 mL of KHS at 37°C and continuously bubbled with a 95% O_2_ to 5% CO_2_ mixture (pH 7.4). Some experiments were performed in endothelium-denuded segments to eliminate the main source of vasoactive substances, including endothelial NO. This avoided possible actions by different drugs on endothelial cells that could lead to misinterpretation of results. Endothelium was removed by gently rubbing the luminal surface of the segments with a thin wooden stick. The segments were subjected to a tension of 0.5 g, which was readjusted every 15 min during a 90-min equilibration period before drug administration. After this, the vessels were exposed to 75 mmol/L KCl, to check their functional integrity. Endothelium removal did not alter the contractions elicited by 75 mmol/L KCl. After a washout period, the presence/absence of vascular endothelium was tested by the ability/inability of 10 µmol/L acetylcholine (ACh) to relax segments precontracted with NA (1 µmol/L).

Vasodilator response to ACh (0.1 nmol/L-10 µmol/L) was tested in endothelium-intact arteries from all experimental groups.

Frequency-response curves to EFS (1, 2, 4, 8 and 16 Hz) were performed in endothelium-intact and endothelium-removed mesenteric segments from all experimental groups. The parameters used for EFS were 200 mA, 0.3 ms, 1–16 Hz, for 30 s with an interval of 1 min between each stimulus, the time required to recover basal tone. A washout period of at least 1 h was necessary to avoid desensitisation between consecutive curves. Two successive frequency-response curves separated by 1-hour intervals produced similar contractile responses. To evaluate the neural origin of the EFS-induced contractile response, the nerve impulse propagation blocker, tetrodotoxin, (TTX, 0.1 µmol/L) was added to the bath 30 min before the second frequency-response curve was performed.

To determine the participation of sympathetic innervation in the EFS-induced response in endothelium-denuded segments from control and BF rats, 1 µmol/L phentolamine, an α-adrenoceptor antagonist, or phentolamine plus 0.1 mmol/L suramin, a non-specific P2 purinergic receptor antagonist, were added to the bath 30 min before performing the frequency-response curve. Additionally, the vasoconstrictor response to exogenous NA (1 nmol/L-10 µmol/L) was tested in segments from both experimental groups.

To study the possible participation of sensitive innervation in EFS-induced response in endothelium-denuded segments from control and BF rats, 0.5 µmol/L CGRP (8–37), a CGRP receptor antagonist, was added to the bath 30 min before performing the second frequency–response curve.

To analyse the participation of NO in the EFS- induced response in endothelium-denuded segments from control and BF rats, 0.1 mmol/L N^ω^-nitro-L-arginine methyl ester (L-NAME), a non-specific inhibitor of nitric oxide synthase (NOS), was added to the bath 30 min before performing the second frequency–response curve. The vasodilator response to the NO donor, diethylamine NONOate, (DEA-NO, 0.1 nmol/L–0.1 mmol/L) was determined in NA-precontracted arteries from both experimental groups.

### Noradrenaline and ATP Release

Endothelium-denuded segments of rat mesenteric arteries from control and BF rats were preincubated for 30 minutes in 5 mL of KHS at 37°C and continuously gassed with a 95% O_2_–5% CO_2_ mixture (stabilisation period). This was followed by two washout periods of 10 min in a bath of 0.4 mL KHS. Then the medium was collected to measure basal release. Next, the organ bath was refilled and cumulative EFS periods of 30 s at 1, 2, 4, 8 and 16 Hz were applied at 1 min intervals. Afterwards, the medium was collected to measure EFS-induced neurotransmitter release. Mesenteric segments were weighed in order to normalise the results.

NA and ATP releases were measured using Noradrenaline Research EIA (Labor Diagnostica Nord, Gmbh and Co., KG, Nordhon, Germany) or an ATP Colorimetric/Fluorometric Assay kit (Abcam, Cambridge, UK). The assays were performed following the manufacturers’ instructions. Results were expressed as ng NA/mL mg tissue, or nmol ATP/mL mg tissue.

### Nitric Oxide Release

NO release was measured using fluorescence emitted by the fluorescent probe 4,5-diaminofluorescein (DAF-2). Endothelium-denuded mesenteric arteries from control and BF rats were subjected to a 60-minute equilibration period in HEPES buffer (in mmol/L: NaCl 119; HEPES 20; CaCl_2_ 1.2; KCl 4.6; MgSO_4_ 1; KH_2_PO_4_ 0.4; NaHCO_3_ 5; glucose 5.5; Na_2_HPO_4_ 0.15; pH 7.4) at 37°C. Arteries were incubated with 2 µmol/L DAF-2 for 30 min. The medium was then collected to measure basal NO release. Once the organ bath was refilled, cumulative EFS periods of 30 s at 1, 2, 4, 8 and 16 Hz were applied at 1 min intervals. Afterwards, the medium was collected to measure EFS-induced NO release. The fluorescence of the medium was measured at room temperature using a spectrofluorometer (LS50 Perkin Elmer Instruments, FL WINLAB Software, Whaltmann, MA, USA) with excitation wavelength set at 492 nm and emission wavelength at 515 nm.

The EFS-induced NO release was calculated by subtracting basal NO release from that evoked by EFS. Also, blank sample measures were collected in the same way from segment-free medium in order to subtract background emission. Some assays were performed in the presence of 0.1 µmol//L TTX or 1 µmol/L 1400W, the specific iNOS inhibitor. The amount of NO released was expressed as arbitrary units/mg tissue.

### Detection of O_2_^.–^

O_2_^.–^ levels were measured using lucigenin chemiluminescence. Endothelium-denuded mesenteric segments from control and BF rats were rinsed in KHS for 30 min, equilibrated for 30 min in HEPES buffer at 37°C, transferred to test tubes that contained 1 mL HEPES buffer (pH 7.4) containing lucigenin (5 µmol/L) and then kept at 37°C. The luminometer was set to report arbitrary units of emitted light; repeated measurements were collected for 5 min at 10 s intervals and averaged. 4,5-Dihydroxy-1,3-benzene-disulphonic acid “Tiron” (10 mmol/L), a cell-permeant, non-enzymatic O_2_^.–^ scavenger, was added to quench the O_2_^.–^dependent chemiluminescence. Also, blank samples were collected in the same way without mesenteric segments to subtract background emission.

### nNOs and P-nNOS Expression

Western blot analysis of nNOS and phosphorylated nNOS (P-nNOS) expression was performed in endothelium-denuded mesenteric segments from control and BF rats, as previously described [22]. For these experiments, we used mouse monoclonal nNOS antibody (1∶1000, Transduction Laboratories), rabbit polyclonal P-nNOS antibody (1∶2000, Abcam, Cambridge, UK), and monoclonal anti-ß -actin-peroxidase antibody (1∶50000, Sigma-Aldrich, Spain). Rat brain homogenates were used as positive control.

### Drugs Used

L -NA hydrochloride, ACh chloride, diethylamine NONOate diethylammonium salt, CGRP (8–37), suramin hexasodium salt, TTX, L -NAME hydrochloride, 1400W, phentolamine, lucigenin, tiron and DAF-2 (Sigma-Aldrich, Madrid, Spain) were used. Stock solutions (10 mmol/L) of drugs were made in distilled water, except for NA, which was dissolved in a NaCl (0.9%)-ascorbic acid (0.01% w/v) solution. These solutions were kept at –20°C and appropriate dilutions were made in KHS on the day of the experiment.

### Data Analysis

The responses elicited by EFS and NA were expressed as a percentage of the initial contraction elicited by 75 mmol/L KCl for comparison between control and BF rats. The relaxation induced by ACh or DEA-NO was expressed as a percentage of the initial contraction elicited by NA (Control: 1070.54±4.6 mg; BF: 1096.92±5.1 mg; after BF: 1054.17±8.5; P>0.05). For concentration-response curves, non-linear regression and E_max_ and –log EC_50_ were performed. Results are given as mean±SEM. Statistical analysis was done by comparing the curve obtained in the presence of the different substances with the previous or control curve by means of repeated measure analysis of variance (ANOVA) followed by Bonferroni post-hoc test, using GraphPad Prism 5.0 software (CA, USA). Some results were expressed as differences of area under the curve (dAUC) of EFS obtained in segments from control and BF animals. AUC were calculated from the individual concentration-response plots. For dAUC, NO, O_2_^.^, ATP and NA release data, the statistical analysis was done using one-way ANOVA followed by Newman-Keuls post-hoc test. P<0.05 was considered significant.

## Results

### Vasomotor Response to KCl

In endothelium-intact mesenteric segments, the vasoconstrictor response to 75 mmol/l KCl was similar in all experimental groups (Control: 1393±89.65 mg; BF: 1445±164.85 mg; After BF: 1412±120.54 mg; P>0.05). Endothelium removal did not alter KCl-induced vasoconstriction (Control: 1348±97.95 mg; BF: 1354±85.77 mg; After BF: 1395±124.46 mg; P>0.05).

### Vasodilator Response to ACh

**Figure 1:**
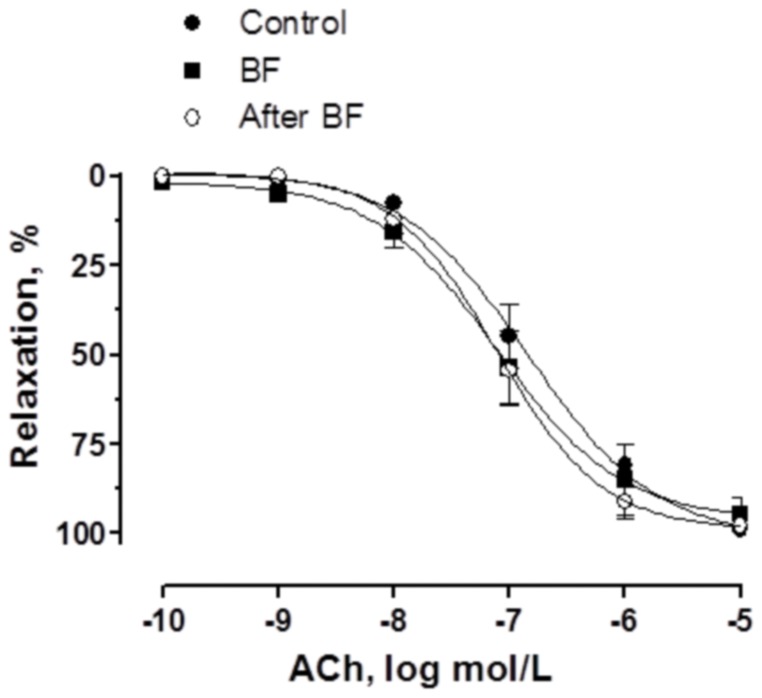
Vasodilator response to ACh. ACh-induced vasodilation in endothelium-intact mesenteric segments from control, breast-feeding (BF) and after BF rats. Results (mean±SEM) were expressed as a percentage of the previous tone elicited by exogenous NA. n = 6 animals each group.

Vasodilator response to ACh was similar in all experimental groups (Figure 1, Table 1).

**Table 1:** E_max_ (%) and log EC_50_ (mmol/L) values of vasodilator responses to ACh in mesenteric arteries from control, BF and after BF female rats.

	Control	BF	After BF
**E_max_**	101.5±6.7	96.20±7.4	98.92±4.5
**−log EC_50_**	6.94±0.2	7.10±0.3	7.10±0.1

Results are expressed as means±S.E.M. n = 6 animals each group.

### Vascular Responses to EFS

**Figure 2:**
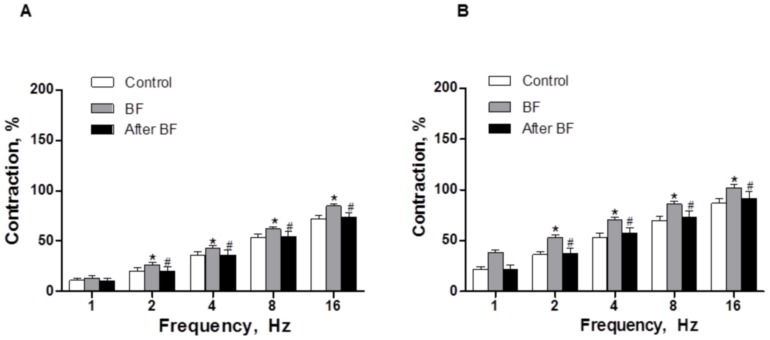
Vasoconstrictor response to EFS. EFS-induced vasoconstriction in endothelium-intact (A) and endothelium-removed (B) mesenteric segments from control, breast feeding (BF) and after BF rats. Results (Mean±S.E.M.) were expressed as a percentage of the initial contraction elicited by KCl. ANOVA P<0.05 Control vs. BF; ANOVA P<0.05 BF vs. After BF in both endothelium intact (A) and endothelium-removed (B) arteries. *P<0.05 vs. control animals at each frequency; #P<0.05 vs. BF group at each frequency (Bonferroni test). n = 10 animals each group.

The application of EFS induced a frequency-dependent contractile response in endothelium-intact mesenteric segments from all experimental groups. This vasoconstriction was greater in segments from BF rats compared to control and after BF animals (Figure 2A). Endothelium removal increased EFS-induced contractile response similarly in segments from all experimental groups (Figure 2B, Table 2). EFS-induced contractions were practically abolished in segments from all experimental groups by the neurotoxin TTX (0.1 µmol/L), indicating the neuronal origin of the factors inducing this response (Results not shown).

**Table 2:** EFS potentiation after endothelium removal.

	1 Hz	2 Hz	4 Hz	8 Hz	16 Hz
**Control**	16.9±3.5	19.2±2.1	22.4±2.3	18.1±2.5	15.7±2.9
**BF**	21.2±3.1	22.4±1.8	24.3±3.4	21.7±2.7	18.3±1.7
**After BF**	17.6±2.9	20.7±1.7	21.9±2.9	19.2±3.1	17.6±2.1

Percentage (%) of potentiation in EFS-induced contraction after endothelium removal. Calculations are performed taking KCl-induced contraction as 100% of the contractile response. Results are expressed as mean ± SEM. n = 10 animals each group.

Since the differences appear only between control and BF rats, and both endothelial and neuronal function are reversed to control situation in after BF rats, we performed the following experiments only in mesenteric segments from control and BF animals.

### Effect of BF on Sympathetic Component of Vascular Responses to EFS

**Figure 3:**
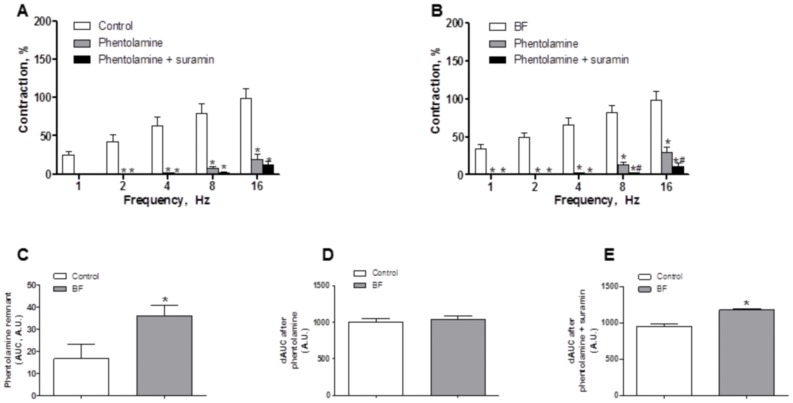
Effect of breast feeding on sympathetic innervation function. Effect of preincubation with 1 µmol/L phentolamine or 1 µmol/L phentolamine plus 0.1 mmol/L suramin on the vasoconstrictor response induced by EFS in endothelium-denuded mesenteric segments from control (A) and breast-feeding (BF) rats (B). Results (Mean±S.E.M.) are expressed as a percentage of the previous contraction elicited by KCl. n = 6 animals each group. ANOVA P<0.05 vs. conditions without phentolamine in both experimental groups. *P<0.05 vs. conditions without phentolamine at each frequency (Bonferroni test). ANOVA P<0.05 phentolamine vs. phentolamine plus suramin in both experimental groups. # P<0.05 phentolamine vs. phentolamine plus suramin at each frequency (Bonferroni test). (C) Representation of remnant vasoconstriction after preincubation with 0.1 µmol/L phentolamine, expressed as area under curve (AUC). *P<0.05 control vs. BF. Differences of area under curve (dAUC) in the absence or presence of 01 µmol/L phentolamine (D) or in the absence or presence of 1 µmol/L phentolamine plus 0.1 mmol/L suramin (E). dAUC values are expressed as arbitrary units. *P<0.05 control vs. BF.

**Figure 4:**
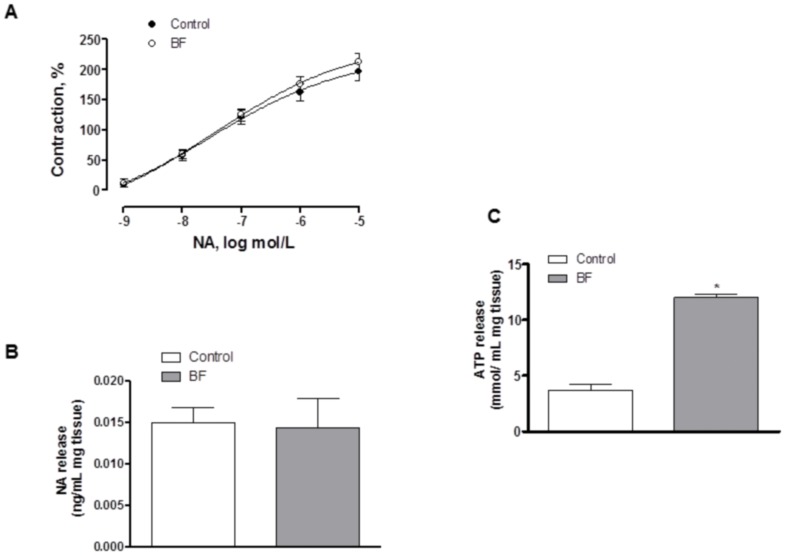
Effect of breast feeding on NA vasoconstriction and release, and ATP release. (A) Vasoconstrictor response to exogenous NA in segments from control and breast-feeding (BF) rats. Results (Mean±S.E.M.) are expressed as a percentage of the previous contraction elicited by KCl. n = 8 animals each group. (B) EFS-induced NA release in mesenteric segments from control and breast-feeding (BF) rats. Results (Mean±S.E.M.) are expressed as ng NA/mL mg tissue. n = 6 animals per group. (C) EFS-induced ATP release in mesenteric segments from control and breast-feeding (BF) rats. Results (Mean±S.E.M.) are expressed as mmol ATP/mL mg tissue. *P<0.05 vs control. n = 7 animals per group.

Preincubation with the α-adrenergic antagonist phentolamine (1 µmol/L) decreased the vasoconstrictor response induced by EFS in segments from both experimental groups to the same extent (Figure 3A, 3B and 3C). In agreement with these results, the contraction elicited by exogenous NA (1 nmol/L-10 µmol/L) was similar in segments from both experimental groups (Figure 4A, Table 3). Additionally, EFS-induced NA release was similar in segments from both experimental groups (Figure 4B).

**Table 3:** E_max_ (%) and log EC_50_ (mmo/L) values of vasoconstrictor response to NA or vasodilator response to DEA-NO in mesenteric arteries from control and BF rats.

	Control		BF	
	E_max_	−log EC_50_	E_max_	–log EC_50_
**NA**	226.3±55.3	7.6±0.8	242.4±45.2	7.37±0.5
**DEA-NO**	100.9±3.4	6.04±0.1	108.1±8.9	5.89±0.2

Results are expressed as means±S.E.M. n = 7–8 animals each group.

Nevertheless, the remnant vasoconstriction induced by EFS after phentolamine preincubation was greater in segments from BF animals. Preincubation with phentolamine plus 0.1 mmol/L suramin, a non-specific P2 purinergic receptor antagonist, decreased EFS-induced contraction in segments from both experimental groups. This decrease was greater in segments from BF animals (Figure 3A, 3B and 3D). In line with this, EFS-induced ATP release was increased in segments from BF rats (Figure 4C).

### Effect of BF on Sensory Component of Vascular Responses to EFS

Preincubation with the CGRP receptor antagonist CGRP (8–37) (0.5 µmol/L) did not alter the EFS-induced contraction in any experimental group (Results not shown).

### Effect of BF on Nitrergic Component of Vascular Responses to EFS

**Figure 5:**
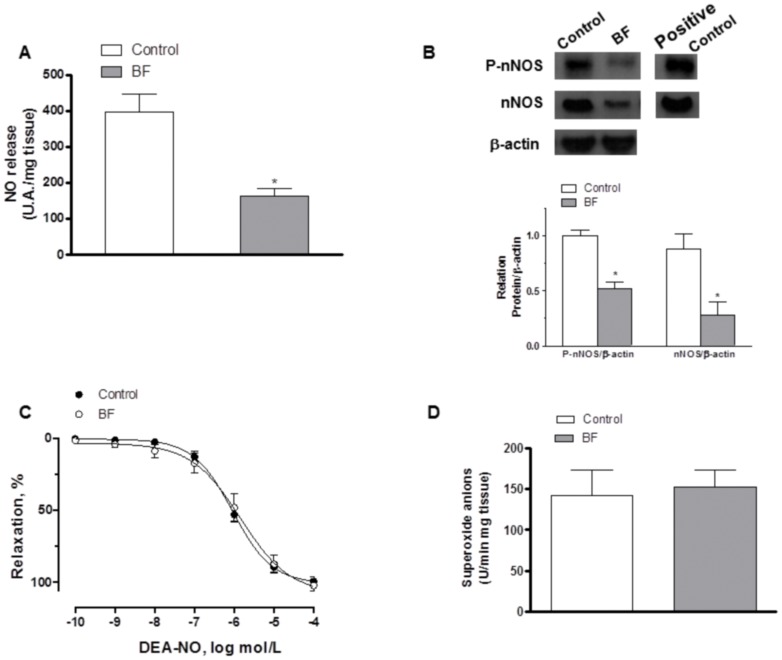
Effect of breast feeding on neuronal NO synthesis and vasodilation. (A) EFS-induced NO release in segments from control and breast-feeding (BF) rats. Results (Mean±S.E.M.) are expressed as arbitrary units (A.U.)/mg tissue. *P<0.05 vs. Control; n = 8 animals per group. (B) Effect of breast feeding (BF) on nNOS and P-nNOS expression. The blot is representative of four separate segments from each group. Rat brain homogenates were used as positive control. Lower panel shows relation between P-nNOS or nNOS expression and ß-actin. Results (Mean±S.E.M.) are expressed as the ratio of the signal obtained for each protein and the signal obtained for ß-actin. *P<0.05 vs. control. (C) Vasodilator response to NO donor DEA-NO in segments from control and breast-feeding (BF) rats. Results (Mean±S.E.M.) are expressed as a percentage of the previous tone elicited by exogenous NA. n = 7 animals each group. (D) Superoxide anion release in mesenteric segments from control and breast-feeding (BF) rats. Results (Mean±S.E.M.) are expressed as chemiluminiscence units (U)/min mg tissue. n = 4 animals per group.

EFS-induced NO release was significantly lower in segments from BF rats (Figure 5A). Preincubation with 0.1 µmol/L TTX abolished EFS-induced NO release, while preincubation with 1 µmol/L of the specific iNOS inhibitor 1400W did not modify it (Results not shown). Both nNOS and P-nNOS expression were diminished in segments from BF rats (Figure 5B). In segments precontracted with NA, vasodilator response elicited by the exogenous NO donor DEA-NO (0.1 nmol/L-10 µmol/L) was similar in mesenteric segments from both experimental groups (Figure 5C, Table 3). Additionally, after subtracting the lucigenin chemiluminescence obtained in the presence of Tiron from that obtained in its absence, superoxide anion formation was similar in segments from both experimental groups (Figure 5D).

**Figure 6:**
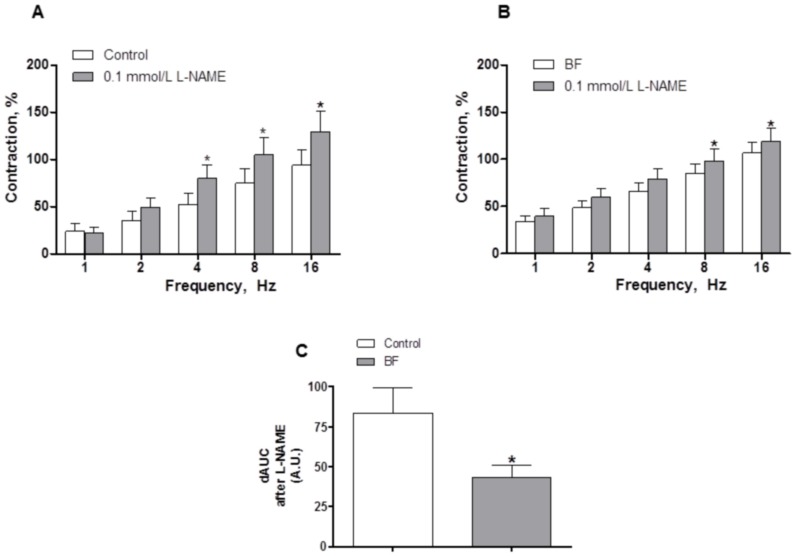
Effect of breast feeding on nitrergic innervation. Effect of preincubation with 0.1 mmol/L L-NAME on the vasoconstrictor response induced by EFS in mesenteric segments from control (A) and breast-feeding (BF) rats (B). Results (Mean±S.E.M.) are expressed as a percentage of the previous contraction elicited by KCl. n = 8 animals each group. ANOVA P<0.05 vs. conditions without L-NAME in both experimental groups. *P<0.05 vs. conditions without L-NAME for each frequency (Bonferroni test). (C) Differences of area under curve (dAUC) in the absence or presence of 01 µmol/L L-NAME. dAUC values are expressed as arbitrary units. *P<0.05.

In line with these results, preincubation with the unspecific NO synthase inhibitor L-NAME (0.1 mmol/L) significantly increased the EFS-contractile response in segments from both experimental groups. This increase was lower in segments from BF rats (Figure 6).

## Discussion

The vasoconstrictor response to EFS is increased by breast feeding and is restored after this physiological process. This increase is endothelium-independent, and is mediated by at least two mechanisms: an increase in ATP release and a decrease in neuronal NO release.

Important differences in vascular reactivity to different agonists have been described in several physiological processes associated to changes in hormone levels. Previous results have been contradictory, since changes as well as no changes have both been reported in the vasoconstrictor response to KCl associated with modifications in sexual hormone levels [23], [24], [25]. In our experimental conditions the vasoconstrictor response to KCl remained unmodified by breast feeding.

When analysing the vasoconstrictor response induced by EFS in endothelium-intact segments, we observed that EFS produced a frequency-dependent contraction in segments from all experimental groups. Mesenteric arteries from breast-feeding rats showed an increased response to EFS. This increase was abolished after breast feeding, and was not attributable to changes in the intrinsic contractile machinery as was demonstrated by the similar vasoconstrictor response to KCl in all experimental groups. Endothelium removal increased vasoconstrictor response to EFS to the same extent in the three experimental groups, indicating that the modulating role of endothelium is not modified by breast feeding. The fact that ACh-induced vasodilation was not modified in any experimental group reinforces this observation. Therefore, these results indicate that the increment observed during breast feeding is due to modifications in perivascular innervation function, as was confirmed by the abolishment of EFS-induced vasoconstriction in the presence of TTX. Additionally, since both endothelial and neuronal function are not different when comparing control and after BF rats, we performed the following experiments only in mesenteric segments from control and BF animals.

Hence, this effect could be associated with changes in neurotransmitter release and/or vasomotor response to the neurotransmitter/s. In order to clarify this aspect, our next objective was to determine possible differences in the participation of sympathetic innervation between control and breast-feeding experimental groups. The participation of sympathetic innervation in splanchnic blood flow adaptation to different situations has been demonstrated previously [26]. Sympathetic innervation releases mainly NA and ATP when electrically stimulated. The results of several studies are inconclusive regarding whether or not sexual hormones affect sympathetic activity [27], [28], [29], but both increases and decreases have been reported during breast feeding [16], [17], [18]. The fact that the α-adrenoceptor antagonist phentolamine significantly diminished the vasoconstrictor response to EFS in mesenteric segments from both experimental groups confirms that this response appears to be mediated mainly by the release of NA from sympathetic nerve terminals. In our study the decreases in EFS vasoconstriction induced by phentolamine, NA release and exogenous NA-induced vasoconstriction were similar in both experimental groups, indicating the non-participation of the adrenergic component from sympathetic innervation in the increased vasoconstrictor response to EFS in breast-feeding rats. It should also be mentioned that in both groups there was a substantial contractile response that was phentolamine-resistant. This remnant vasoconstriction was higher in segments from breast feeding animals. In mesenteric arteries we have previously demonstrated that this remnant vasoconstriction is due to ATP release from sympathetic nerve endings [30]. Based on this information, we analysed EFS-induced contraction after simultaneous preincubation with phentolamine plus the non-specific P_2_ purinergic receptor antagonist suramin. In these conditions, the contractile response to EFS was reduced in both experimental groups, but the reduction was greater in segments from BF than in control rats, indicating a higher ATP participation in this response during BF. In line with these results, EFS-induced ATP release was higher in mesenteric segments from BF than control rats. Taken together, these results indicate that the greater EFS-induced vasoconstriction observed in mesenteric segments from breast-feeding rats might be due to an increase in sympathetic function through increased ATP release. However, the participation of other kinds of innervation cannot be ruled out.

We have previously reported that CGRP released from sensory innervation does not participate in the vasoconstrictor response to EFS in our experimental conditions [22], [30]. However, we have also demonstrated that in several situations, such as hypertension and diabetes [11], [15], [33], CGRP does begin to be functional. Since sex hormones have been shown to modulate CGRP release [31], we analyzed the possibility that breast feeding might produce changes in sensory innervation function in rat mesenteric innervation. The non-modification of EFS-induced vasoconstriction obtained after preincubation with CGRP receptor antagonist CGRP (8–37) indicates that breast feeding does not modify the participation of sensory innervation-derived CGRP in vasomotor response to EFS.

The participation of neuronal NO released from perivascular nitrergic innervation in the EFS-induced response has been exhaustively studied by our group [13], [22], [30], [32]. In the experimental conditions presented in this study, the involvement of neuronal NO in the EFS-induced response is demonstrated by the increased response to EFS in segments from both experimental groups after preincubation with the unspecific NOS inhibitor L-NAME. This increase was greater in segments from breast-feeding rats, indicating that this condition could induce differences in the neuronal NO pathway. Previous studies have reported increased nitrite and nitrate levels in breast-feeding mother’s serum [19], suggesting an increase in NO release, which could be originated from different sources, such as endothelium and innervation. However, in our experimental conditions, EFS-induced NO release was significantly decreased by breast feeding. NO production in neural tissue can have two sources: nNOS or iNOS [22], [30], [32], [34]. The fact that preincubation with TTX abolished EFS-induced NO release in segments from both groups of rats, and that preincubation with the specific iNOS inhibitor 1400W did not alter NO release, confirms the neural origin and rules out the inducible origin of the NO. Since we have previously demonstrated in this vascular bed that NO released from nerve endings is synthesized through nNOS [22], [30], our next objective was to determine whether this decrease was due to alterations in nNOS expression and/or activation. We observed by Western blot analysis that both nNOS and P-nNOS expression were diminished in mesenteric segments from breast-feeding rats compared to controls, indicating that the local decrease of neuronal release in breast-feeding rats is caused by decreased nNOS expression and activation.

Increased ROS generation has been described specially at the end of gestation and the beginning of lactation [20]. Thus, the involvement of ROS in the vascular response cannot be ruled out, since it would alter *the* neuronal NO metabolism and consequently affect the bioavailability of neuronal NO. Superoxide anion release was similar in segments from both control and breast-feeding rats. Additionally, the vasodilator response to the NO donor DEA-NO was similar in segments from both experimental groups confirming that the diminished function of the nitrergic innervation is due to a decreased neuronal NO release and not to changes in the vasodilator response and/or metabolism of neuronal NO.

In conclusion, breast feeding increases the contractile response induced by EFS in mesenteric arteries and this increase appears to be mainly mediated by a decrease in the neuronal NO release mediated by decreased nNOS and P-nNOS expression. Additionally, sympathetic innervation function is increased through increased ATP release. These alterations might have relevance when mothers develop hypertension during the breast feeding period.
